# 0.5 V and 0.43 pJ/bit Capacitive Sensor Interface for Passive Wireless Sensor Systems

**DOI:** 10.3390/s150921554

**Published:** 2015-08-28

**Authors:** Andoni Beriain, Iñigo Gutierrez, Hector Solar, Roc Berenguer

**Affiliations:** CEIT and Tecnun (University of Navarra), Manuel de Lardizabal 15, Donostia 20018, Spain; E-Mails: ingutierrez@ceit.es (I.G.); hsolar@ceit.es (H.S.); rberenguer@ceit.es (R.B.)

**Keywords:** capacitive-sensor interface, low-power sensor interface, period modulation, pressure sensor

## Abstract

This paper presents an ultra low-power and low-voltage pulse-width modulation based ratiometric capacitive sensor interface. The interface was designed and fabricated in a standard 90 nm CMOS 1P9M technology. The measurements show an effective resolution of 10 bits using 0.5 V of supply voltage. The active occupied area is only 0.0045 mm${}^{2}$ and the Figure of Merit (FOM), which takes into account the energy required per conversion bit, is 0.43 pJ/bit. Furthermore, the results show low sensitivity to PVT variations due to the proposed ratiometric architecture. In addition, the sensor interface was connected to a commercial pressure transducer and the measurements of the resulting complete pressure sensor show a FOM of 0.226 pJ/bit with an effective linear resolution of 7.64 bits. The results validate the use of the proposed interface as part of a pressure sensor, and its low-power and low-voltage characteristics make it suitable for wireless sensor networks and low power consumer electronics.

## 1. Introduction

Sensor usage is undergoing a great growth, due in particular to its inclusion in new generation wireless sensor networks (WSN) and consumer electronics. The main challenge to their implementation is reducing the power consumption of the sensor nodes, which limits the battery life of the sensor devices [1]. In the specific case of passive WSN, a reduction in sensor power consumption together with low supply voltages that enable the use of high efficiency power harvesting modules [2] would make it possible to increase the communication range of the sensors.

Therefore, there is great interest in the development of low-power and low-voltage sensor systems. With this objective, novel architectures based on capacitive transducers and time to digital conversion have been reported recently [3,4,5]. In these novel architectures, capacitive transducers are preferred over resistive transducers due to their high relative sensitivity, low temperature dependence and virtually negligible power consumption [6]. These novel sensor interfaces are based on period modulation (PM) or pulse-width modulation (PWM) instead of using a signal conditioning stage and analog-to-digital converters (ADC). This way, the capacitive value of the transducer modulates the width/period of a pulse generated through a capacitance-to-time converter such an oscillator or similar. The transducer capacitance dependent pulses are subsequently digitized using a simple time-to-digital converter (*i.e.*, a digital counter). These interfaces are quite flexible and the resolution can be easily traded for measurement time by counting the duration of multiple output periods [4]. In addition, they may have very low power-consumption and active area [3,4,5] in comparison with traditional architectures.

This work describes an energy-efficient and ultra low-power, low-voltage PWM based capacitive-sensor interface. The paper is organized as follows. In Section 2, the operating principle and design considerations of the interface are presented. Section 3 discusses the circuit implementation and the experimental results. Finally, the paper is concluded in Section 4.

## 2. Operating Principle

In this section the architecture of the proposed interface and the key points of the design are discussed.

### 2.1. Architecture

**Figure 1:**
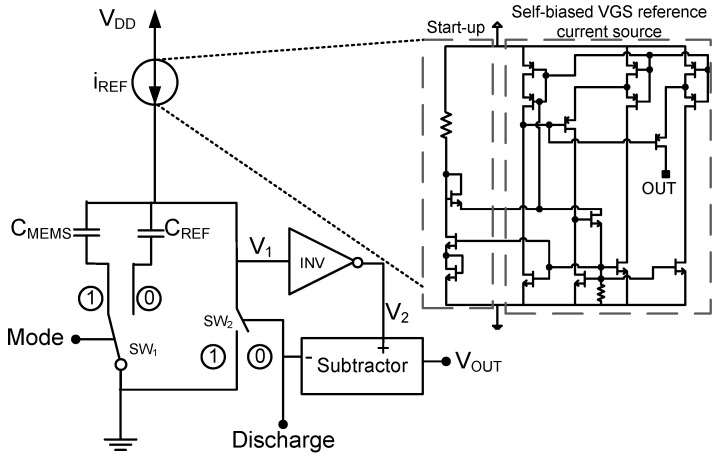
Proposed architecture.

The architecture of the proposed interface, presented in Figure 1, is based on a relaxation oscillator formed by an inverter (INV), two capacitors (C${}_{\text{MEMS}}$ and C${}_{\text{REF}}$), a constant current source (i${}_{\text{REF}}$) and two switches (SW${}_{1}$ and SW${}_{2}$). In addition to the C${}_{\text{MEMS}}$ capacitor that represents the capacitive sensor transducer, the interface also measures the value of an integrated C${}_{\text{REF}}$ capacitor. This allows ratiometric measurements of C${}_{\text{MEMS}}$ against the C${}_{\text{REF}}$, simplifying the sensor calibration and making it robust against PVTs.

**Figure 2:**
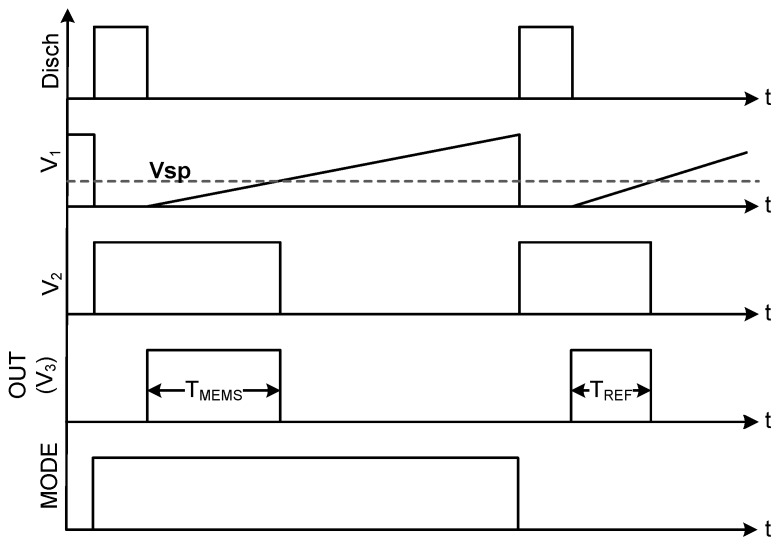
Signal diagram during a measurement.

As Figure 2 shows, the capacitance to time conversion starts when the discharge signal (“Discharge”) goes from “0” to “1”. At this moment, node “V${}_{1}$” is shortcut to ground through switch “SW${}_{2}$”. Switch “SW${}_{1}$”, which is controlled by the “Mode” signal, selects which capacitor is discharged. When the “Discharge” signal falls to “0”, “SW${}_{2}$” is opened and the conversion starts. At that moment, the capacitor selected by the “Mode” signal starts charging with the current i${}_{\text{REF}}$ and voltage “V${}_{1}$” rises. When voltage “V${}_{1}$” is above the inverter switching point (V${}_{\text{SP}}$), “V${}_{2}$” goes down and stays down until a discharge pulse arrives again to the “SW${}_{2}$” switch.

The output of the capacitance-to-time conversion (T) is the length of the pulse that results from subtracting the “Discharge” signal from the “V${}_{2}$” signal. This length is defined by the time required by “V${}_{1}$” to rise from “0” to “V${}_{\text{SP}}$” and it is obtained by using the capacitance charging formula presented in Equation (1). Equation (2) shows the pulse width (T) obtained after rearranging and solving the equation for this specific case.

(1) $$i_{\text{REF}}=C\frac{dV_{1}}{dt}$$

(2) $$T=t_{\left(V_{1}=V_{\text{SP}}\right)}-t_{\left(V_{1}=0\right)}=\frac{C*V_{\text{SP}}}{i_{\text{REF}}}$$

The capacitance-to-time conversion of both capacitors is done in series and controlled by the “Mode” signal. As the conversion of both capacitors is done using the same current source and comparator, the effect of PVT errors in both generated pulses is similar and the size of the circuit is reduced. Once both C${}_{\text{MEMS}}$ and C${}_{\text{REF}}$ have been converted into T${}_{\text{MEMS}}$ and T${}_{\text{REF}}$, the time-to-digital conversion is performed using two counters and a fast oscillator with frequency F${}_{\text{OSC}}$ to sample the length of both pulses. The digitalized value for C${}_{\text{MEMS}}$ and C${}_{\text{REF}}$ are described by Equations (3) and (4).

(3) $$D_{\text{MEMS}}=\frac{C_{\text{MEMS}}F_{\text{OSC}}V_{\text{SP}}}{i_{\text{REF}}}$$

(4) $$D_{\text{REF}}=\frac{C_{\text{REF}}F_{\text{OSC}}V_{\text{SP}}}{i_{\text{REF}}}$$

The main advantage of this architecture is that as D${}_{\text{MEMS}}$ and D${}_{\text{REF}}$ are generated and sampled using the same current source, inverter and oscillator, the average values of V${}_{\text{SP}}$, i${}_{\text{REF}}$ and oscillator frequency can be considered as a first order approximation to be equal during both pulses generation. Moreover, if the pulses are generated one right after the other, the effect of PVT variations are equivalent and proportional in D${}_{\text{MEMS}}$ and D${}_{\text{REF}}$. Therefore, as Equation (5) shows, if a ratiometric system is used to measure C${}_{\text{MEMS}}$ against C${}_{\text{REF}}$, the ratio between both measured pulse lengths (D${}_{\text{MEMS}}$ and D${}_{\text{REF}}$) is the ratio between the capacitor values and does not depend on any other design parameter or their PVT variations. Therefore, as C${}_{\text{REF}}$ has a constant value and the value of C${}_{\text{MEMS}}$ depends on the physical magnitude to be measured through the transducer, this magnitude can be easily estimated from Equation (5).

(5) $$R=\frac{D_{\text{MEMS}}}{D_{\text{REF}}}=\frac{C_{\text{MEMS}}}{C_{\text{REF}}}$$

### 2.2. PVT Variations

As Equation (5) shows, the output of the interface depends uniquely in the ratio between two capacitors, C${}_{\text{REF}}$ and C${}_{\text{MEMS}}$. As C${}_{\text{MEMS}}$ is the value to be estimated with the interface, the PVT variations that need to be compensated for at the output are C${}_{\text{REF}}$ variations.

As far as process variations goes, a single-point calibration is enough to determine C${}_{\text{REF}}$ nominal value and compensate for its effects in the output ratio.

First order effects caused by voltage variations, current variations and inverter threshold variations, are compensated for at architecture level due to the ratiometric output and the current source that isolates the capacitor from the voltage supply.

Last but not least, temperature variations depend on the implemented C${}_{\text{REF}}$ capacitor and its characteristics. Integrated capacitors have small-medium temperature coefficient and in some applications its effect could be negligible. However, in high temperature range or high accuracy applications, a temperature calibration may be necessary to achieve the desired accuracy in the measurements.

### 2.3. Parasitic Capacitance

The parasitic capacitance at node “V${}_{1}$” affects directly to Equation (5) and might introduce offset and slope deviations. However, it was observed that after parasitic post-layout extraction this parasitic capacitor was in the order of femto-farads, small in comparison with C${}_{\text{REF}}$ and C${}_{\text{MEMS}}$ that are around 10 pF. Also test set-up parasitic capacitance was observed to be very small when compared to C${}_{\text{REF}}$ and C${}_{\text{MEMS}}$. Therefore, its effect in the equation is not critical and may be neglected in many applications or compensated for partially with the previously mentioned single-point process calibration. In any case, the parasitic capacitor will be constant during the operation of the interface. This means that, it does not affect to the linearity of the interface. Therefore, in the worst case, when parasitic capacitance is comparable to C${}_{\text{REF}}$ and C${}_{\text{MEMS}}$, a traditional two-point process calibration would be enough to totally compensate for its effects, even in most critical applications. In the characterization of the interface, no influence of this parasitic capacitor was observed.

### 2.4. Resolution

In order to obtain a resolution of N bits, the difference in the MEMS counter output (D${}_{\text{MEMS}}$) between the minimum value of C${}_{\text{MEMS}}$ (C${}_{\text{ZS}}$) and the maximum value of C${}_{\text{MEMS}}$ (C${}_{\text{FS}}$) conversion should be greater than 2${}^{\text{N}}$. Therefore, the sampling oscillator period (T${}_{\text{OSC}}$) must satisfy the condition shown in Equation (6), where T${}_{\text{MEMS}}$(C${}_{\text{FS}}$) and T${}_{\text{MEMS}}$(C${}_{\text{ZS}}$) refer to the pulse width correspondent to C${}_{\text{FS}}$ and C${}_{\text{ZS}}$ conversion respectively; and ΔT${}_{\text{MEMS}}$ refers to the variation range of T${}_{\text{MEMS}}$.

(6) $$T_{\text{OSC}}<\frac{T_{\text{MEMS}}\left(C_{\text{FS}}\right)-T_{\text{MEMS}}\left(C_{\text{ZS}}\right)}{2^{N}}=\frac{\Delta T_{\text{MEMS}}}{2^{N}}$$

### 2.5. Current Source

The current source is an important block of the design as the minimum supply voltage is limited by this block. Taking into account the importance of working at low operation voltages in wireless passive applications to increase harvesting efficiency [2], it is critical to design the current source for very low voltage operation. As shown in Figure 1, a self-biased VGS reference current source with a current mirroring technique based on [7] to ensure very high output impedance when working at voltages as low as 0.5 V has been implemented.

### 2.6. Model Validation

The ratiometric output presented in Equation (5) is based on the assumptions that inverter and subtractor delay is neglectable in comparison with T${}_{\text{MEMS}}$ and that V${}_{\text{SP}}$ is constant during the conversion of two subsequent pulses with input capacitance in the range between C${}_{\text{ZS}}$ and C${}_{\text{FS}}$. In order to check that these assumptions are correct and to validate Equation (5) the following conditions must be met.

#### 2.6.1. Delay

**Figure 3:**
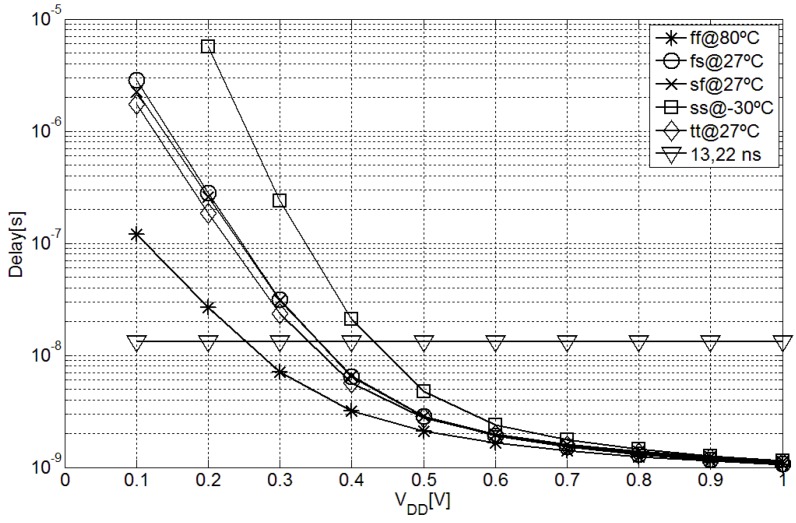
Cumulative inverter and subtractor delay in different fabrication corners with different supply voltages.

The most intuitive criteria for neglecting the inverter and subtractor cumulative delay effect at the output of the capacitance-to-time converter is to assure that it is below the sensitivity (LSB) of the time-to-digital converter. As detailed in section III, in order to obtain a resolution of 10 bits for the implemented MEMS transducer a fast oscillator of 75.6 MHz is necessary. Therefore, if the maximum delay for every fabrication corner and supply voltage is below its period (13.22 ns), this effect could be neglected. Figure 3 shows the post-layout simulated cumulative delay for different supply voltages and fabrication corners. As shown, for supply voltages of 0.5 V or higher the effect of the delay is below 1 LSB, and therefore could be considered negligible.

#### 2.6.2. Switching Voltage

Ideally, the switching point (V${}_{\text{SP}}$) of the inverter is constant and equal to V${}_{\text{DD}}/2$. However, it usually changes when the input voltage slope ($\alpha =\frac{dV}{dT}$) changes.

(7) $$\alpha =\frac{dV}{dT}=\frac{i_{\text{REF}}}{C}$$

In this design, because *α* depends on C and i${}_{\text{REF}}$ (Equation (7), V${}_{\text{SP}}$ may change during different C${}_{\text{MEMS}}$ measurements, adding nonlinearities to the measurements. Equations (8) and (9) show T${}_{\text{MEMS}}$ generated as result of C${}_{\text{ZS}}$ and C${}_{\text{FS}}$ conversion with constant V${}_{\text{SP}}$. The only difference between both equations is the k factor between capacitor values (C${}_{\text{FS}}$=C${}_{\text{ZS}}$*k).

(8) $$T_{\text{MEMS}}\left(C_{\text{ZS}}\right)=\frac{C_{\text{ZS}}*V_{\text{SP}}}{i_{\text{REF}}}=\frac{V_{\text{SP}}}{\alpha }$$

(9) $$T_{\text{MEMS}}\left(C_{\text{FS}}\right)=\frac{C_{\text{FS}}*V_{\text{SP}}}{i_{\text{REF}}}=k\frac{C_{\text{ZS}}*V_{\text{SP}}}{i_{\text{REF}}}=k\frac{V_{\text{SP}}}{\alpha }$$

Equations (10) and (11) show the same expressions but include the influence of V${}_{\text{SP}}$ on the output time of the converter when measuring C${}_{\text{MEMS}}$. In order to ensure that this variation doesn’t affect the linearity of the interface, the design needs to meet the condition expressed in Equation (12), where the influence of V${}_{\text{SP}}$ on the output time is less than one LSB.

(10) $$T_{\text{MEMS}}^{\prime }\left(C_{\text{ZS}}\right)=\frac{C_{\text{ZS}}*V_{\text{SP\_ZS}}}{i_{\text{REF}}}=\frac{V_{\text{SP\_ZS}}}{\alpha }$$

(11) $$T_{\text{MEMS}}^{\prime }\left(C_{\text{FS}}\right)=\frac{C_{\text{FS}}*V_{\text{SP\_FS}}}{i_{\text{REF}}}=k\frac{V_{\text{SP\_FS}}}{\alpha }$$

(12) $$|T_{\text{MEMS}}^{\prime }\left(C_{\text{FS}}\right)-k*T_{\text{MEMS}}^{\prime }\left(C_{\text{ZS}}\right)|<\frac{T_{\text{MEMS}}^{\prime }\left(C_{\text{ZS}}\right)\left(k-1\right)}{2^{N}}$$

Substituting Equations (10) and (11) in Equation (12) and developing the expression, the maximum allowable V${}_{\text{SP}}$ variation is obtained in Equation (13), as a function of the k and the number of bits of the interface.

(13) $$|V_{\text{SP\_FS}}-V_{\text{SP\_ZS}}|<\frac{V_{\text{SP\_ZS}}\left(k+1\right)}{k*2^{N}}$$

Equation (13) has been validated at simulation level with N equal to 10 and K, C${}_{\text{ZS}}$ and C${}_{\text{FS}}$ corresponding to a commercial capacitive pressure transducer [8]. The results show that as long as T${}_{\text{MEMS}}$(C${}_{\text{ZS}}$) is bigger than 20.44 *μ*s, Equation (13) is satisfied and the V${}_{\text{SP}}$ variations effect could be neglected as its influence on the capacitance-to-time converter is below one LSB.

## 3. Experimental Section

**Figure 4:**
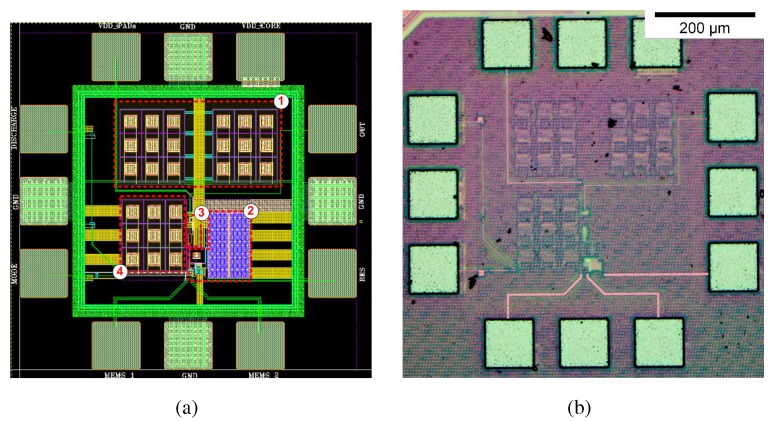
(**a**) Layout plot and (**b**) photograph of the chip.

The capacitive sensor interface based on capacitance-to-time conversion was fabricated in standard 90 nm CMOS 1P9M technology. Figure 4 shows the layout and a microphotograph of the fabricated chip, with an active area of 0.045 mm${}^{2}$. In the layout the different parts of the design are identified: (1) Supply-to-ground capacitors, (2) capacitance-to-time converter, (3) output buffer and (4) integrated C${}_{\text{REF}}$ capacitor. The time to digital conversion has not been included in this implementation in order to compare the design with the state of the art (Table 1).

The design has been optimized to work with a commercial capacitive pressure transducer [8] that acts as C${}_{\text{MEMS}}$. The transducer shows an output variation between 7.82 pF and 11.79 pF with an input range between 30 kPa and 120 kPa. Regarding C${}_{\text{REF}}$, it is a standard integrated MIM cap with a value of 9 pF.

### 3.1. Results

#### 3.1.1. Capacitance-to-Time Conversion

**Figure 5:**
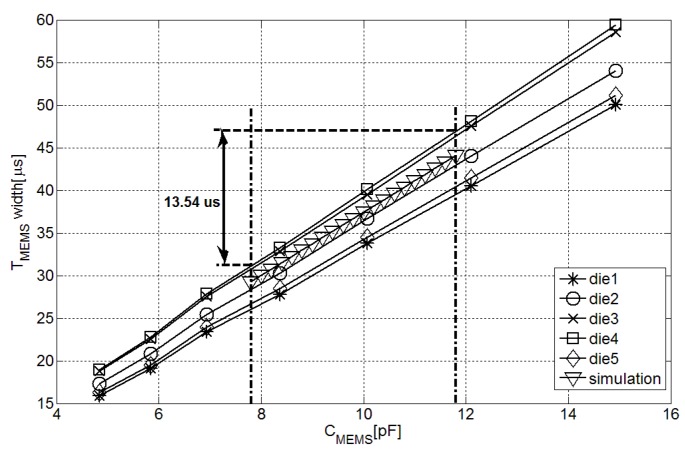
Measurement-pulse length in the fabricated interfaces with different C${}_{\text{MEMS}}$.

Five dies were connected to different capacitors with a supply voltage of 0.5 V to check the operating principle of the interface. Figure 5 shows the evolution of T${}_{\text{MEMS}}$ against C${}_{\text{MEMS}}$.

This graph demonstrates the interface conversion capability within the 4.5 pF to 15.2 pF capacitance dynamic range. However, the interface has been optimized to work with [8] commercial pressure transducer, which has a capacitance range between 7.82 pF and 11.79 pF. The minimum $▵T_{\text{MEMS}}$ or dynamic range corresponding to this capacitance range is 13.54 *μ*s, so according to Equation (6) a fast oscillator of 75.6 MHz will be sufficient in the following time-to-digital converter to ensure a 10 bits resolution. According to our measurements, inside the operation range of [8] the sensor performance in terms of noise effect, average current consumption and minimum supply voltage is homogeneous.

The fabrication process variations are the cause of the interchip offset variation presented in Figure 5 due to changes in i${}_{\text{REF}}$ and transistor speed. The same effect was reported in T${}_{\text{REF}}$ measurements. This way, when C${}_{\text{MEMS}}$ is equal to 12.1 pF, the measured variations in the pulse length between dies are ±8.53% in the case of T${}_{\text{MEMS}}$ and ±8.18% in the case of T${}_{\text{REF}}$.

**Figure 6:**
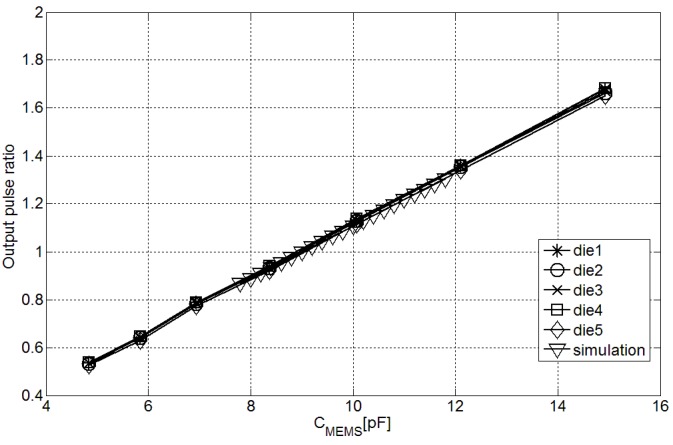
Output ratio in the fabricated interfaces with different C${}_{\text{MEMS}}$.

According to the operating principle of the architecture, the differences due to i${}_{\text{REF}}$ and transistors parameter variations should be compensated when the ratio between T${}_{\text{MEMS}}$ and T${}_{\text{REF}}$ is applied. Figure 6 shows this premise. Without any calibration the output ratio compensates these effects and the interchip variation of the measurements is reduced to ±0.98%, which is caused by C${}_{\text{REF}}$ process variations and can be compensated by a single-point calibration.

**Figure 7:**
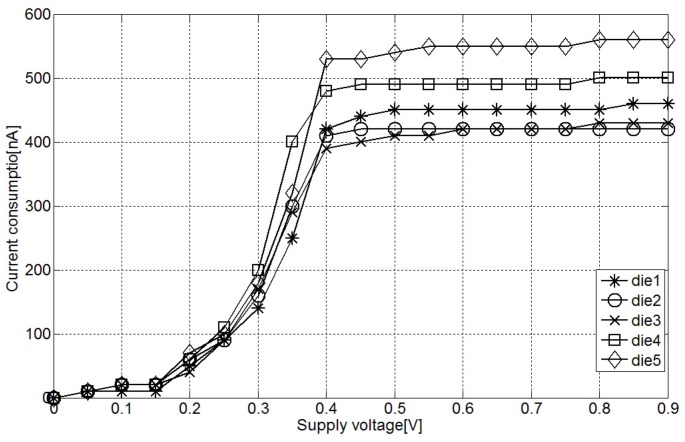
Measured DC current consumption at different supply voltages.

In order to evaluate the supply voltage variation effect in the measurements, first, the static current consumption of the interface has been evaluated with different supply voltages. As Figure 7 shows, the DC current is stable with voltages above 0.4 V, once that the minimum supply voltage of the current source is reached. Considering that minimum supply voltage is one of the desired characteristics for the interface, it has been set at 0.5 V, leaving a margin of 100 mV from the current source minimum.

**Figure 8:**
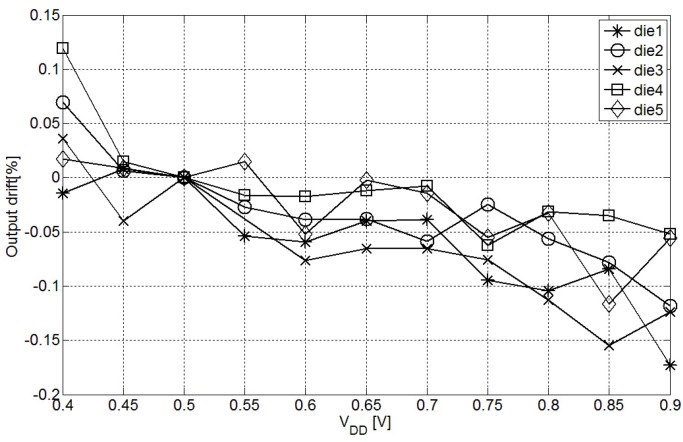
Supply voltage variation effect in the output ratio.

In Figure 8 the effect of voltage variations in the interface output is evaluated. The ratiometric architecture and high output impedance current source compensates for first order voltage variation effects in the measurement, reporting a variation around ±0.1% in the output within 0.4 V to 0.6 V supply voltage range.

Regarding the measurement time, the system requires a maximum time of 47 μs to convert C${}_{\text{MEMS}}$ and 35.4 μs to convert C${}_{\text{REF}}$. The discharge pulses are set to 10 μs each, so the complete measuring time required by the interface is 102.4 μs with a measured average current consumption of 0.882 μA.

**Figure 9:**
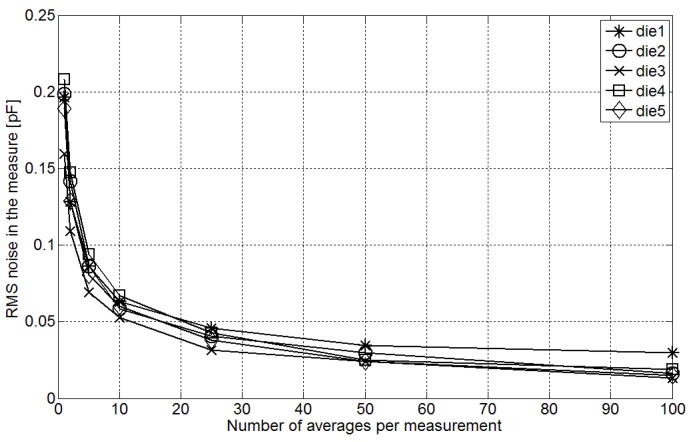
RMS noise in the measurements when converting a 10 pF C${}_{\text{MEMS}}$.

Next, the noise characterization of the fabricated converters, which has a thermal origin, was carried out. Figure 9 shows the result of the converter output ratio (T${}_{\text{MEMS}}$ /T${}_{\text{REF}}$) noise characterization in five different dies measuring a 10 pF C${}_{\text{MEMS}}$. The RMS error due to noise in the conversion is presented, depending on the number of averaged samples per measurement. It can be observed that the RMS noise error is reduced when the number of averaged samples per measurement rises. If the average of 10 samples is used to obtain a measurement, the value of the Effective Resolution (ER) corresponding to the RMS noise is above 10 bits. This increases the measuring time of the converter to 1.02 ms.

#### 3.1.2. Pressure-to-Time Conversion

In order to test the behavior of the capacitance-to-time interface working as part of a pressure sensor, it was connected to a MEMS pressure-to-capacitance transducer [8]. Taking into account that the MEMS transducer requires a three-point calibration the objective of this characterization is to evaluate the error generated in the pressure measurements that cannot be compensated for with the three-point calibration.

**Figure 10:**
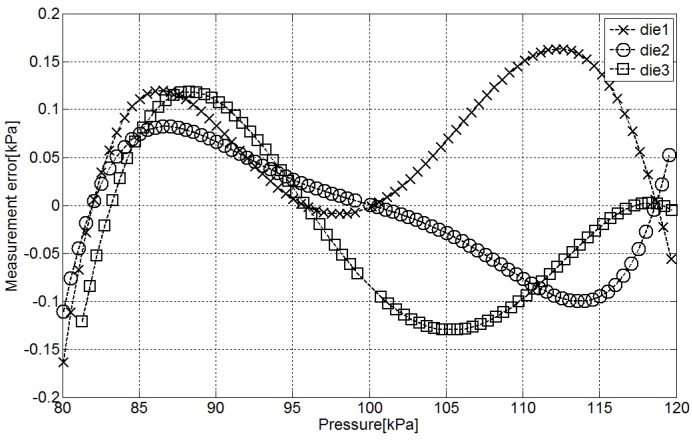
Error in the pressure estimation with the fabricated pressure sensor.

The difference between pressure measured in a pressure reference (GE DPI 620) and same pressure estimated through the proposed interface is presented in Figure 10. The mean accuracy of the pressure sensor due to the accumulative nonlinearities of the MEMS pressure transducer and the fabricated sensor interface after three-point calibration is approximately ±0.1 kPa in the range between 80 kPa and 120 kPa. This way, the conversion inaccuracy is ±0.25% of the measuring range which corresponds to an effective linear resolution around 7.6 bit with an LSB of 0.2 kPa. According to the measurements presented in Figure 9 the ER of the interface is around 9 bit with a single sample per measurement, enough with this linear resolution. Therefore, a measuring time of 102 μs with an average current consumption of 0.882 μA will be sufficient to perform the pressure measurements with 7.6 bits of resolution.

### 3.2. Discussion

A performance summary and a comparison with other state-of-the-art capacitive-sensor interfaces are shown in Table 1. The reported figure-of-merit (FOM) normalizes the energy consumption to the resolution. It is derived from the well-known FOM for evaluating general purpose ADCs [4], and is calculated as in Equation (14), where I${}_{\text{AV}}$, T${}_{\text{MEAS}}$ and ER correspond to the average current consumption, measurement time and Effective Resolution respectively.

(14) $$FOM\left(J/bit\right)=\frac{I_{\text{AV}}V_{\text{DD}}T_{\text{MEAS}}}{2^{\text{ER}}}$$

**Table 1:** Comparison of reported capacitive sensor interfaces.

Ref.	Type	Tech	Act. Area	Input	Supply	Current	Meas.	Eff. Res.	Out	FOM
		(μm)	(mm${}^{2}$)	Cap. (pF)	(V)	Cons.	Time.	(bit)		
ISCC’14 [9]	SAR ADC	0.18	0.49	2.5∖75.3	1.2–0.9	160 nA	4 ms	13.3	Digital	0.063 pJ
TCASII’11 [10]	ΣΔ	0.35	0.048	–0.5∖0.5	3.3	436 μA	0.128 ms	11	Digital	90 pJ
A-SSCC’11 [11]	ΣΔ	0.16	0.25	0.4∖1.2	1.8	5.85 μA	10 ms	13	Time	13 pJ
ESSCIRC’11 [5]	PM	0.13	0.0725	6∖6.3	0.3	0.9 μA	1 ms	6.1	Time	3.9 pJ
JSSC’12 [4]	PM	0.35	0.51	6.8	3.3	64 μA	7.6 ms	15	Time	49 pJ
ESSCIRC’08 [6]	PWM	0.32	0.528	0.5∖0.76	3	28 μA	0.033 ms	8	Time	10.8 pJ
TIM’12 [3]	PWM	0.35	0.09	2.5∖2.82	3	18 μA	0.04 ms	9.3	Time	3.4pJ
**This work**	PWM	0.09	0.045	10	0.5	0.882μA	1.02 ms	10	Time	0.43 pJ

A minimum FOM together with a minimum supply voltage and a minimum active area are the desired characteristics for wireless passive sensor systems. As shown in Table 1, where the proposed capacitance sensor interface is compared with the state of the art, the proposed design performs extremely well in these three characteristics, allowing a FOM of 0.43 pJ/bit, a supply voltage of 0.5 V an area of 0.045 mm${}^{2}$ to be obtained. Regarding the area, the proposed circuit is the smallest. The supply voltage is the second smallest, only improved by [5], which reports and outstanding supply voltage of 0.3 V but has a noisy output with only 6.1 bits of ER.

As far as conversion efficiency goes (FOM), the presented design is the second most efficient only improved by [9]. The main strength of [9] is an array of nine capacitors that is adjusted in each measurement and acts as reference capacitor. This technique allows the circuit to cover a huge input capacitance range (2.5 pF to 75 pF) without loosing linearity, increasing the resolution of the sensor up to 13.3 bits. This high resolution together with a very low current consumption leads to a very low FOM. However, the implementation of the capacitor array requires area 10 times larger than the one used in this work.

Therefore, it can be concluded that the presented design has a remarkable overall behavior in terms of supply voltage, design size and FOM. These good features are also validated in the complete pressure sensor. In this case, as a single sample per measurement (no averaging) is enough to ensure a resolution of 7.64 bits, the energy per measurement is reduced to 45.06 pJ and the FOM to only 0.226 pJ/bit.

In order to complete the presented capacitance to time interface, a low power and high efficiency time to digital converter is necessary. In [12] a compatible converter is presented which reports a supply voltage of 0.6 V and a power consumption of 0.53 μW. The combination of these two devices would form an ultra low voltage and low power pressure sensor.

## 4. Conclusions

Given the great interest in the development of low-power and low-voltage sensor systems for WSN and consumer electronics, this paper presented an ultra low-power and low-voltage pulse-width modulation based capacitive sensor interface. The system is based on a simple relaxation oscillator that generates a pulse whose width depends on the value of a capacitive MEMS transducer. In addition, the interface includes an integrated reference capacitor that is also converted to time in order to perform ratiometric measurements of the MEMS transducer and reduce PVT variation effects.

The interface has been designed and fabricated in standard 90 nm CMOS 1P9M technology and measurement results show improved efficiency and reduced chip area relative to reported interfaces based on time conversion. In order to validate the suitability of the interface as part of a pressure sensor it has been successfully connected to a commercial pressure transducer. The results demonstrate the suitability of the proposed architecture for WSN and low-power consumer electronics, and they reinforce the use of time-conversion based sensor interfaces over traditional ADCs in these applications.

## References

[1] Harrop P., Das R. (2012). Wireless Sensor Networks (WSN) 2012-2022: Forecasts, Technologies, Players. The New Market for Ubiquitous Sensor Networks (USN).

[2] Bergeret E., Gaubert J., Pannier P., Gaultier J. (2007). Modeling and Design of CMOS UHF Voltage Multiplier for RFID in an EEPROM Compatible Process. IEEE Trans. Circuits Syst. II Express Briefs.

[3] Sheu M.L., Hsu W.H., Tsao L.J. (2012). A Capacitance-Ratio-Modulated Current Front-End Circuit with Pulsewidth Modulation Output for a Capacitive Sensor Interface. IEEE Trans. Instrum. Meas..

[4] Tan Z., Shalmany S., Meijer G., Pertijs M. (2012). An Energy-Efficient 15-Bit Capacitive-Sensor Interface Based on Period Modulation. IEEE J. Solid-State Circuits.

[5] Danneels H., Coddens K., Gielen G. A fully-digital, 0.3V, 270 nW capacitive sensor interface without external references. Proceedings of the ESSCIRC (ESSCIRC).

[6] Bruschi P., Nizza N., Dei M. A low-power capacitance to pulse width converter for MEMS interfacing. Proceedings of the 34th European Solid-State Circuits Conference (ESSCIRC 2008).

[7] Tanguay L.F., Sawan M., Savaria Y. A very-high output impedance current mirror for very-low voltage biomedical analog circuits. Proceedings of the IEEE Asia Pacific Conference on Circuits and Systems, (APCCAS 2008).

[8] (2010). Capacitive Pressure Sensor 1.2 BAR SCB10H-B012FB Datasheet.

[9] Ha H., Sylvester D., Blaauw D., Sim J.Y. 12.6 A 160nW 63.9 fJ/conversion-step capacitance-to-digital converter for ultra-low-power wireless sensor nodes. Proceedings of the 2014 IEEE International Solid-State Circuits Conference Digest of Technical Papers (ISSCC).

[10] Shin D.Y., Lee H., Kim S. (2011). A Delta Sigma Interface Circuit for Capacitive Sensors with an Automatically Calibrated Zero Point. IEEE Trans. Circuits Syst. II Express Briefs.

[11] Tan Z., Daamen R., Humbert A., Souri K., Chae Y., Ponomarev Y., Pertijs M. A 1.8 V 11 uW CMOS smart humidity sensor for RFID sensing applications. Proceedings of the 2011 IEEE Asian Solid State Circuits Conference (A-SSCC).

[12] Jimenez-Irastorza A., Beriain A., Sevillano J., Rebollo I., Berenguer R. A 0.6 V and 0.53 uW nonius TDC for a passive UHF RFID pressure sensor tag. Proceedings of the 2012 IEEE International Conference on RFID-Technologies and Applications (RFID-TA).

